# Effectiveness of preoperative cognitive behavioral therapy for patients undergoing lumbar spine fusion surgery: A systematic review focusing on patient-reported outcomes

**DOI:** 10.1007/s10143-026-04329-3

**Published:** 2026-05-21

**Authors:** Ali Haider Bangash, Rose Fluss, Sertac Kirnaz, Liza Belman, Alexander Alexandrov, Victoria Cao, Ananth S. Eleswarapu, Mitchell S. Fourman, Jonathan D. Krystal, Yaroslav Gelfand, Saikiran G. Murthy, Reza Yassari, Rafael De la Garza Ramos

**Affiliations:** 1 Department of Neurosurgery, Montefiore Medical Center, Albert Einstein College of Medicine, 3316 Rochambeau Ave, Bronx, NY 10467 USA; 2 Department of Orthopedic Surgery, Montefiore Medical Center, Albert Einstein College of Medicine, Bronx, NY USA

**Keywords:** Lumbar spine fusion surgery, Cognitive behavioral therapy, Patient-reported outcome, Oswestry Disability Index, EQ-5D index, Prehabilitation

## Abstract

To examine implementation approaches and effectiveness of preoperative cognitive behavioral therapy (CBT) on patient-reported outcomes following lumbar fusion. We systematically searched PubMed/Medline, Cochrane Database of Systematic Reviews, and Epistemonikos (inception to December 15, 2024) for studies investigating preoperative CBT in patients scheduled for lumbar fusion. Primary and secondary outcomes were postoperative disability (Oswestry Disability Index, ODI) and health-related quality of life (EQ-5D), respectively, at 3 and 6 months. Meta-analysis employed Inverse Variance Random Effects Standardized Mean Difference models to compare the change from baseline. From a total of 182 studies, three RCTs comprising 307 patients (mean age: 50 years; 58% female) met the inclusion criteria. Meta-analysis revealed no statistically significant differences between CBT and control groups in ODI change from baseline at 3 months (SMD 0.04 [95% CI: -0.81 to 0.88]; *p* = 0.93; I² = 92%) or 6 months (SMD − 0.17 [95% CI: -0.53 to 0.19]; *p* = 0.36; I² = 58%). Similarly, no significant differences emerged for EQ-5D at 3 months (SMD 0.09 [95% CI: -0.27 to 0.46]; *p* = 0.62; I² = 59%) or 6 months (SMD − 0.56 [95% CI: -1.49 to 0.36]; *p* = 0.23; I² = 93%). Current evidence does not support preoperative CBT as broadly effective for unselected lumbar fusion candidates. However, substantial heterogeneity tempers the validity of conclusions across surgical settings. Targeted psychological interventions for high-risk patients (catastrophizing, anxiety, depression) within comprehensive prehabilitation programs warrant consideration.

**Supplementary Information:**

The online version contains supplementary material available at 10.1007/s10143-026-04329-3.

## Introduction

Lumbar fusion surgery represents a significant healthcare intervention. Approximately 200,000 fusions are performed annually in the United States alone, primarily for degenerative conditions, spondylolisthesis, and chronic low back pain refractory to conservative management [1]. Despite the technological advancements in minimally invasive surgical techniques, patient-reported outcomes remain variable, with approximately 13% of patients reporting dissatisfaction following surgery [2]. Psychological factors have emerged as critical determinants of surgical outcomes, with preoperative psychological distress, including depression, anxiety, and pain catastrophizing, consistently associated with poorer functional recovery, increased pain intensity, and reduced quality of life postoperatively [3, 4].

The psychological burden experienced by lumbar fusion candidates manifests in multiple domains, with studies indicating up to 1 in 5 patients demonstrating psychological distress preoperatively [5], and only approximately 1 in 5 patients having no history of psychiatric ailment [6]. Pain catastrophizing is characterized by rumination, magnification, and helplessness regarding pain. It has been particularly implicated in suboptimal surgical outcomes, with higher preoperative catastrophizing scores correlating with greater postoperative pain intensity and functional disability [7]. Similarly, preoperative depression and anxiety have demonstrated robust associations with prolonged opioid use, delayed return to work, and increased healthcare utilization following surgery [8, 9]. Despite these established relationships, standardized approaches to addressing psychological factors before surgery remain notably absent from relevant surgical pathways [10].

Cognitive Behavioral Therapy (CBT) represents a structured, evidence-based psychological intervention that addresses maladaptive thought patterns and behaviors associated with chronic pain and psychological distress.^11^ By targeting catastrophic thinking, fear-avoidance behaviors, and activity pacing, CBT has been shown to reduce pain intensity and improve function across diverse chronic pain populations [12]. The theoretical rationale for preoperative CBT centers on modifying psychological risk factors before surgery to optimize postoperative recovery [13]. Preliminary evidence from other surgical patient groups, including those undergoing total joint arthroplasty and cardiac surgery, suggests that preoperative psychological interventions may reduce postoperative pain, anxiety, and length of hospital stay while improving functional outcomes as well as patient satisfaction [14, 15].

Despite promising findings in other surgical domains, the effectiveness of preoperative CBT specifically for patients undergoing lumbar spine fusion surgery remains unclear [16, 17]. Furthermore, no systematic meta-analytical synthesis of evidence has comprehensively evaluated the implementation approaches and effectiveness of preoperative CBT in this population. This systematic review aimed to address this knowledge gap by examining the methods of preoperative CBT for patients scheduled to be managed with lumbar spine fusion and assessing its impact on critical patient-reported outcomes, including postoperative disability and health-related quality of life.

## Materials and methods

### Search strategy

This systematic review was carried out in accordance with the Preferred Reporting Items for Systematic Review and Meta-Analysis (PRISMA) guidelines [18], with a prospective study protocol (PSP) guiding the objectives, search strategy, and planned analyses developed and subsequently adopted rigorously. Since this study was a systematic review of previously published research, IRB approval was not required (Clinical trial number: not applicable). Three independent reviewers (A.H.B., R.F., and S.K.) performed search strategy implementation, screening, data extraction, quality assessment, and meta-analysis, with disagreements resolved through discussion and, when necessary, consultation with the senior author (R.D.G.R.).

A literature search was performed on December 15, 2024 using PubMed/Medline, Cochrane Database of Systematic Reviews (CDSR), and Epistemonikos in accordance with the said PSP, specifically looking for studies exploring the impact of preoperative CBT on patient-reported outcomes of lumbar spine fusion surgery. The search strategy included the terms “Lumbar Vertebrae”[MeSH Terms], “Spinal Fusion”[MeSH Terms], and “Cognitive Behavioral Therapy”[MeSH Terms]. It is summarized in ‘*Supplementary Materials*’. Furthermore, the reference lists of eligible studies were manually searched for additional publications.

### Objectives and endpoints

Our objectives were: (1) To explore the implementation protocols of preoperative CBT adopted for patients scheduled for lumbar spine fusion; and (2) To assess the impact of preoperative CBT on postoperative patient-reported outcomes.

The primary endpoint was the postoperative change in the degree of disability as measured using the Oswestry Disability Index (ODI) level 3 months and 6 months after surgery when compared to the preoperative baseline. The secondary endpoint was the change in the postoperative health-related quality of life measured using the EQ-5D index level 3 months and 6 months after surgery.

### Study selection

The cohort of eligible articles was reviewed with consideration of abstracts and full-texts as required. Studies were included if they met the following inclusion criteria: (1) Patients scheduled for lumbar spine fusion surgery, irrespective of aetiology, were the primary patient population; (2) Preoperative CBT was implemented; (3) The aforementioned primary, secondary and/or tertiary endpoint(s) were reported in the context of preoperative CBT; and (4) Case series, case-control studies, retrospective or prospective cohort studies as well as randomised-controlled trials (RCTs) published from database inception until December 15, 2024.

Studies were excluded based on methodological and content-specific considerations. Methodologically, we excluded non-empirical publications (review articles, editorials, commentaries, and conference abstracts without full-text availability) to ensure data completeness and quality. Case reports and studies with fewer than 5 participants were planned to be excluded due to limited generalizability and high risk of selection bias. Non-English publications were excluded due to translation resource constraints, which represents an acknowledged limitation of our review.

Content-specific exclusions focused on studies where: (1) lumbar fusion was performed as a secondary or adjunctive procedure rather than the primary intervention; (2) psychological interventions lacked explicit cognitive-behavioral theoretical frameworks or standardized protocols; (3) outcome measures focused exclusively on perioperative variables (such as length of stay or anesthesia requirements) without assessing the patient-reported outcomes central to our research questions; and (4) studies examining only postoperative psychological interventions, as our focus was specifically on preoperative optimization. Studies with mixed surgical populations were planned to be included only if lumbar fusion-specific data could be extracted separately.

Data extraction was performed in accordance with the pre-determined “Characteristics of studies” table. Variables of interest included author and year as well as study characteristics (study location, study design, study setting). Patient demographics (sample size, mean age, gender distribution, race, ethnicity, household income, insurance status, education level, and employment status) were also extracted. CBT particulars (intervention timing, mode of delivery, and intervention content) were inferred. The timing, mode of delivery and content of the control intervention, where available, were also extracted.

Surgical indication, intent, and approach were recorded. Patient-reported outcomes as included in our primary and secondary endpoints were collected. Study limitations recognized risk of bias, and the concluding direction of findings were also extracted.

### Quality assessment and risk of bias

The Revised Cochrane risk-of-bias tool for randomized trials (RoB2) was adopted to measure the methodologic quality and risk of bias of the included RCTs [19]. The RoB2 analysis allowed for the assessment of the overall risk of bias stratified into 5 domains: (1) Randomization process; (2) Deviations from the intended interventions; (3) Missing outcome data; (4) Measurement of the outcome; and (5) Selection of the reported result [19].

### Statistical analysis

An exploratory data analysis was performed. Categorical variables were expressed as percentages of the total, and continuous variables were expressed as mean (with standard deviation) or median (with range). Where the mean values with confidence intervals were reported, the embedded calculator in RevMan software (v. 5.4.1; Cochrane, London, England) was used to calculate the SD. Where median and interquartile range values were provided, an online calculator was used (http://vassarstats.net/median_range.html*)* to calculate mean with SD based on the formulae developed by Hozo SP et al. [20]. Inverse Variance Random Effects Standardized Mean Difference models were developed to compare respective change in ODI (primary endpoint) and EQ-5D index (secondary endpoint) from baseline to 3 months and 6 months after surgery. The I^2^ statistic was used to comment on statistical heterogeneity [21]. Egger’s and Begg’s tests were used to evaluate for publication bias. The RevMan software (v. 5.4.1; Cochrane, London, England) and MedCalc software (v. 20.215; MedCalc Software Ltd, Ostend, Belgium) were used for statistical analysis.

## Results

**Fig. 1:**
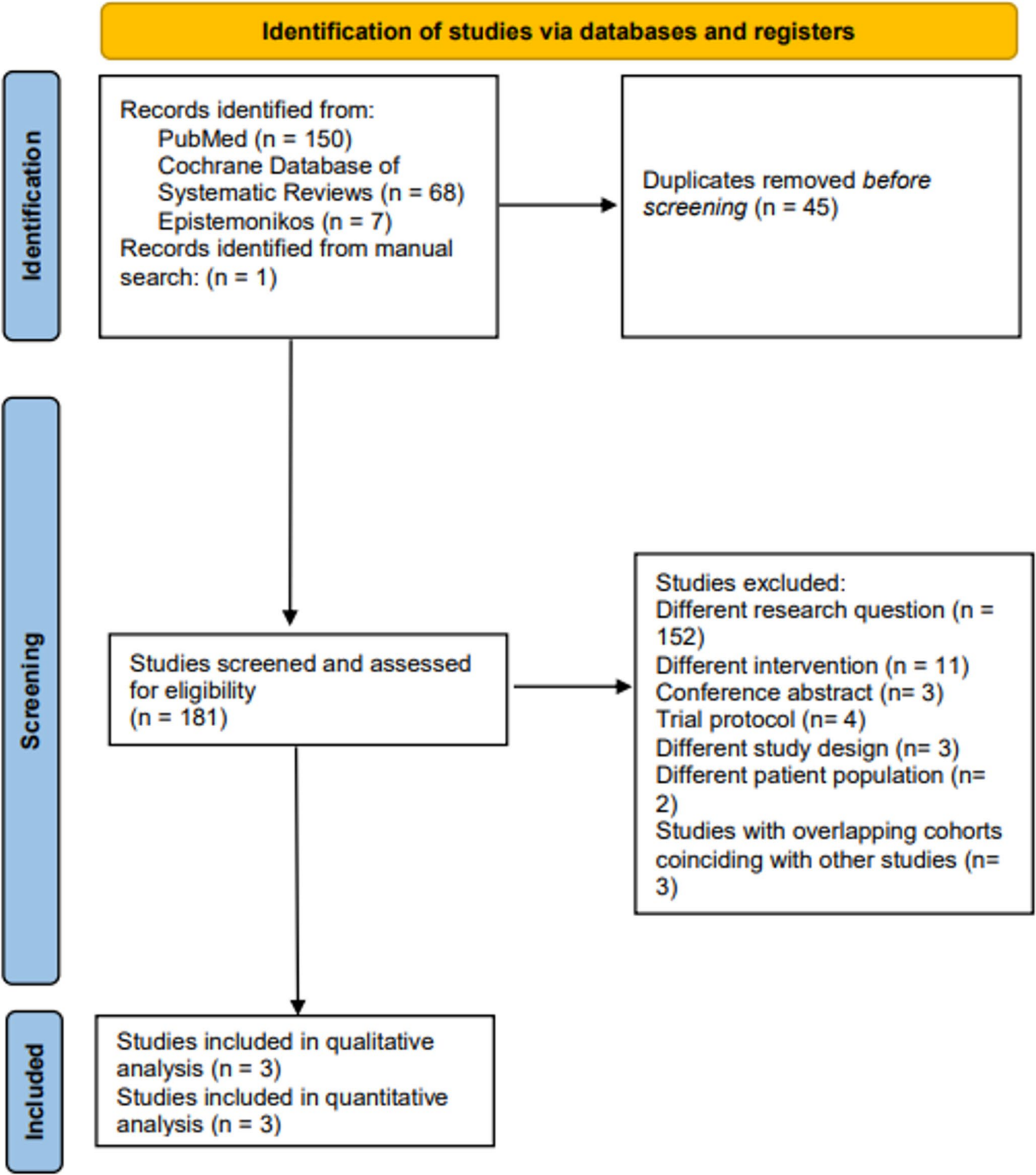
PRISMA flow-chart illustrating search, selection, and inclusion of studies exploring the impact of preoperative cognitive behavioral therapy on patient-reported outcomes following lumbar spine fusion surgery

A search carried out using the PubMed/Medline, CDSR, and Epistemonikos databases yielded 225 articles. One study was obtained after manual evaluation of the reference lists of the included articles. After removing 43 duplicates, the full-texts of 182 studies were screened for eligibility. Three studies were included in our final review [22–24]. The underlying causes for study exclusion are indicated in the PRISMA flow diagram (Fig. 1). The complete list of excluded studies is shared in the ‘*Supplementary Materials*’.

### General patient demographics and studies characteristics

The three included studies were published between 2016 and 2019. They reported on a total of 307 patients with a mean age of 50 years and 58% (*n* = 178) female sex (Table 1). All included studies were RCTs in design, with an evidence level of Level I: Therapeutic Study. 67% (*n* = 2) studies were performed in Denmark [23, 24], and 33% (*n* = 1) studies were performed in Sweden [22]. None of the studies reported the race, ethnicity, household income, or insurance status of the patients. The reported education levels of the study participants varied, with the Lotzke H et al. and Strøm J et al. trials providing detailed breakdowns showing a range from elementary school to university level education (Table 1) [22, 23]. Employment status data was limited, with studies focusing on sick leave or work status (employed versus unemployed) at baseline, rather than reporting specific job titles or industries.

**Table 1:** Demographics of the included studies exploring the impact of preoperative cognitive behavioral therapy on patient-reported outcomes for patients scheduled for lumbar spine fusion surgery

Study cohort	Study setting	Total patients (male and female)	Mean age	Education Level	Employment Status data	Indication for surgery	Surgical Procedure(s)
Lotzke H et al., 2019^**⁂**^ [22]	2 private spine clinics and 1 university hospital	118 patients 55 male (47%) and 63 female (53%)	Mean: 45.7 (± 8.3) years	-Elementary School: 7 patients (5.9%) -High School: 51 patients (43.2%) -University: 42 patients (35.6%) -Vocational: 17 patients (14.4%)	-No sick leave: 75 patients (63.6%) -Full-time sick leave: 25 patients (21.2%) -Part-time sick leave: 16 patients (13.6%)	-Interventebral disc herniation, foraminal stenosis, or isthmic spondylolisthesis	-Instrumented posterior fusion: 103 patients (87.3%) -Instrumented anterior interbody fusion: 1 patient (0.8%) -Instrumented combined posterior and interbody fusion: 4 patients (3.4%)
Strøm J et al., 2019 [23]	Orthopedic spine department of a university hospital	99 patients 35 male (35%) and 64 female (65%)	Mean: 54 (29–79) years	-Basic education (early childhood education, primary education, and lower secondary education): 22 patients (22%) -Secondary education (upper secondary education): 73 patients (74%) -Higher education (post-secondary non tertiary education, short-cycle tertiary education, bachelors or equivalent, masters or equivalent, doctoral or equivalent level): 4 patients (4%)	-Employed: 33 patients (33%) -Sick leave/disability pension/unemployed: 39 patients (39%) -Retirement/student: 27 patients (27%)	-Spondylolisthesis: 35 patients (35%) -Degenerative disease: 64 patients (65%)	-Posterolateral fusion: 94 patients (95%) -Transforminal interbody fusion: 5 patients (5%)
Rolving N et al., 2016 [24]	Orthopedic departments of a university hospital and a general hospital	90 patients 39 male (43%) and 51 female (57%)	Mean: 50.1 (28–64) year	Not reported	-Employed: 47 patients (52%) -Unemployed: 22 patients (24%) -Disability pension: 14 patients (16%) -Early retirement: 7 patients (8%)	-Spondylolisthesis: 23 patients (25.5%) -Degenerative disease: 67 patients (74.5%)	-Posterolateral fusion: 53 patients (59%) -Transforminal interbody fusion: 36 patients (40%) -Uninstrumented: 1 patient (1%)

**⁂** Education level data not reported for 1 patient and employement status data not reported for 2 patients. Moreover, 10 patients were not surgically intervened due to undefined reasons

Spondylolisthesis and degenerative disease were the most common surgical indications, with posterolateral fusion the most commonly used approach (Table 1). 67% (*n* = 2) studies excluded patients having an underlying psychiatric ailment [23, 24].

### Methodological quality assessment

**Fig. 2:**
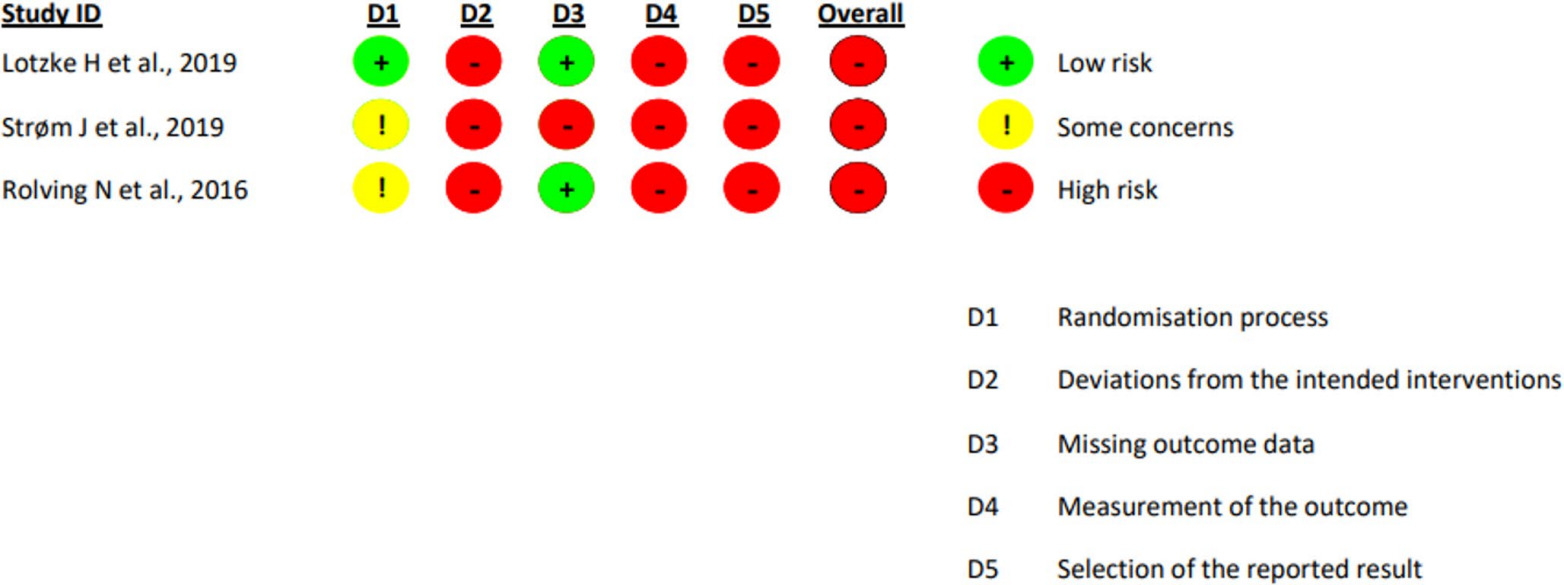
The revised Cochrane risk-of-bias tool for randomized trials (RoB2) analysis of the included studies exploring the impact of preoperative cognitive behavioral therapy on patient-reported outcomes following lumbar spine fusion surgery

All included studies had a ‘high’ overall risk of bias when assessed using the RoB2 tool, primarily due to deviations from intended interventions and high uncertainty in outcomes measurement **(**Fig. 2). 67% (2 of 3) studies had a low risk of bias for the ‘Missing Outcome Data’ domain [22, 24], whereas all three included studies exhibited high uncertainty in outcome measurement (Fig. 2) [22–24].

### Preoperative cognitive behavioral therapy intervention

Diverse approaches were used to deliver preoperative psychological interventions for patients scheduled for lumbar spine fusion, ranging from traditional in-person CBT to web-based platforms (Table 2). Rolving N et al. implemented a structured group-based CBT protocol comprising six 3-hour sessions (four preoperative, two postoperative) delivered by a multidisciplinary team [24, 25]. The intervention was standardized using provider training and manual-guided delivery, with regular monitoring to ensure protocol adherence [24, 25].

**Table 2:** Interventions reported in the included studies exploring the impact of preoperative cognitive behavioral therapy on patient-reported outcomes for patients scheduled for lumbar spine fusion surgery

Study cohort	CBT group intervention timing	Mode of Delivery of CBT group intervention	CBT group intervention Content	Control group intervention timing	Mode of Delivery of Control group intervention	Control group intervention Content
Lotzke H et al., 2019 [22]	Pre-op + post-op	- Four 1-hour in-person sessions at a spine clinic before surgery - One 30-minute telephone session 2 weeks after surgery	- Predefined structure with specific aims - Cognitive-behavioral techniques - Delivered by an experienced physical therapist with additional training in CBT	Pre-op only	- Single session with a physical therapist at the surgical clinic	- Information about postoperative mobilization routine - Introduction to core exercise program - Encouragement to stay active and perform recommended exercises before surgery
Strøm J et al., 2019 [23]	Pre-op + post-op	- Web-based intervention (w-SPIINA) - Access through designated website − 15-minute introduction at baseline - PLUS the control group intervention	- Animated information (16 min, 17 sequences) - Internet support group (ISG) - Diary for tracking pain and physical ability - Designed to reduce anxiety, catastrophic thoughts, and misconceptions - Peer support from former patients as facilitators	Pre-op + post-op	− 2-hour joint session 1 to 5 weeks before surgery - Supervised physical rehabilitation starting at 12 weeks postoperatively	- Information on operative and anesthetic procedure, course of treatment, medication, postoperative training, and restrictions - Oral presentation supported by slides and written handouts - Delivered by nurses, physiotherapists, and occupational therapists
Rolving N et al., 2016 [24]	Pre-op + post-op	- Six 3-hour group sessions − 4 sessions preoperatively − 2 sessions postoperatively - PLUS the control group intervention	- Interaction of cognition and pain perception - Coping strategies - Pacing principles - Ergonomic directions - Return to work - Details about surgical procedure - Delivered by a multidisciplinary team (psychologist, occupational therapist, physiotherapist, social worker, spine surgeon, and previously operated patient) - Standardized sessions with some flexibility	Pre-op + post-op	- Preoperative information sessions - Postoperative rehabilitation starting 12 weeks after surgery	- Information about the operation, anesthetic procedure, medication, postoperative rehabilitation, and physical restrictions - Delivered by operating surgeon, nurses, physiotherapists, and occupational therapists − 8 weeks of postoperative supervised exercise (individual or group) at local rehabilitation centers or physiotherapy clinics

Lotzke H et al. adopted an individualized approach, offering four 1-hour preoperative sessions and one postoperative telephone follow-up [22]. Their intervention was delivered by a single, highly qualified physical therapist with extensive clinical experience and specialized CBT training [22]. This contrasted with the innovative web-based intervention (w-SPIINA) adopted by Strøm J et al., which utilized digital technology to deliver CBT principles through animated content, an internet support group, and interactive features [23]. The w-SPIINA platform incorporated 16-minute animated sequences divided into 17 chapters, covering the entire treatment journey from preparation through post-surgical rehabilitation [23].

Each study employed distinct mechanisms to enhance patient engagement and learning (Table 2). The protocol employed by Rolving N et al. emphasized direct therapeutic interaction and peer support through group sessions [24, 25], while Lotzke H et al. focused on personalized, one-on-one therapeutic relationships [22]. The digital approach explored by Strøm J et al. uniquely addressed accessibility barriers through animated narratives designed to accommodate varying health literacy levels, while incorporating non-catastrophic imagery in an attempt to modify beliefs and behaviors of patients [23]. The platform also featured an internet support group facilitated by former patients and a self-monitoring diary [23].

Moreover, adherence to and completion of preoperative CBT interventions emerged as a clinical consideration across studies. Rolving N et al. reported that of the 49 patients allocated to CBT that completed the intervention, 30% (*n* = 15) patients attended all four sessions, 43% (*n* = 21) patients attended three sessions, and 27% (*n* = 13) patients attended two sessions [25, 26]. Common reasons for non-compliance included inability to take time off work, pain during travel to the hospital, and personal circumstances [25, 26]. Strøm J et al. noted high engagement with their web-based intervention, with 90% of patients accessing the interactive support group and 48% being active contributors [23].

### Primary endpoint

**Fig. 3:**
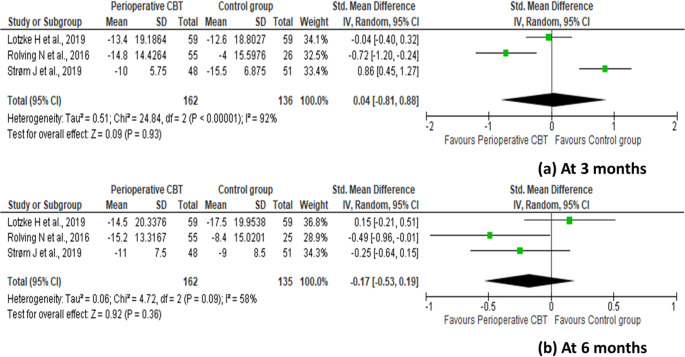
Forest plot with standardized mean difference (with 95% confidence intervals) for the change in the postoperative disability (Oswestry Disability Index) from baseline to (**a**) 3 months postoperatively and (**b**) 6 months postoperatively after lumbar spine fusion surgery, comparing preoperative cognitive behavioral therapy with standard care

**Fig. 4:**
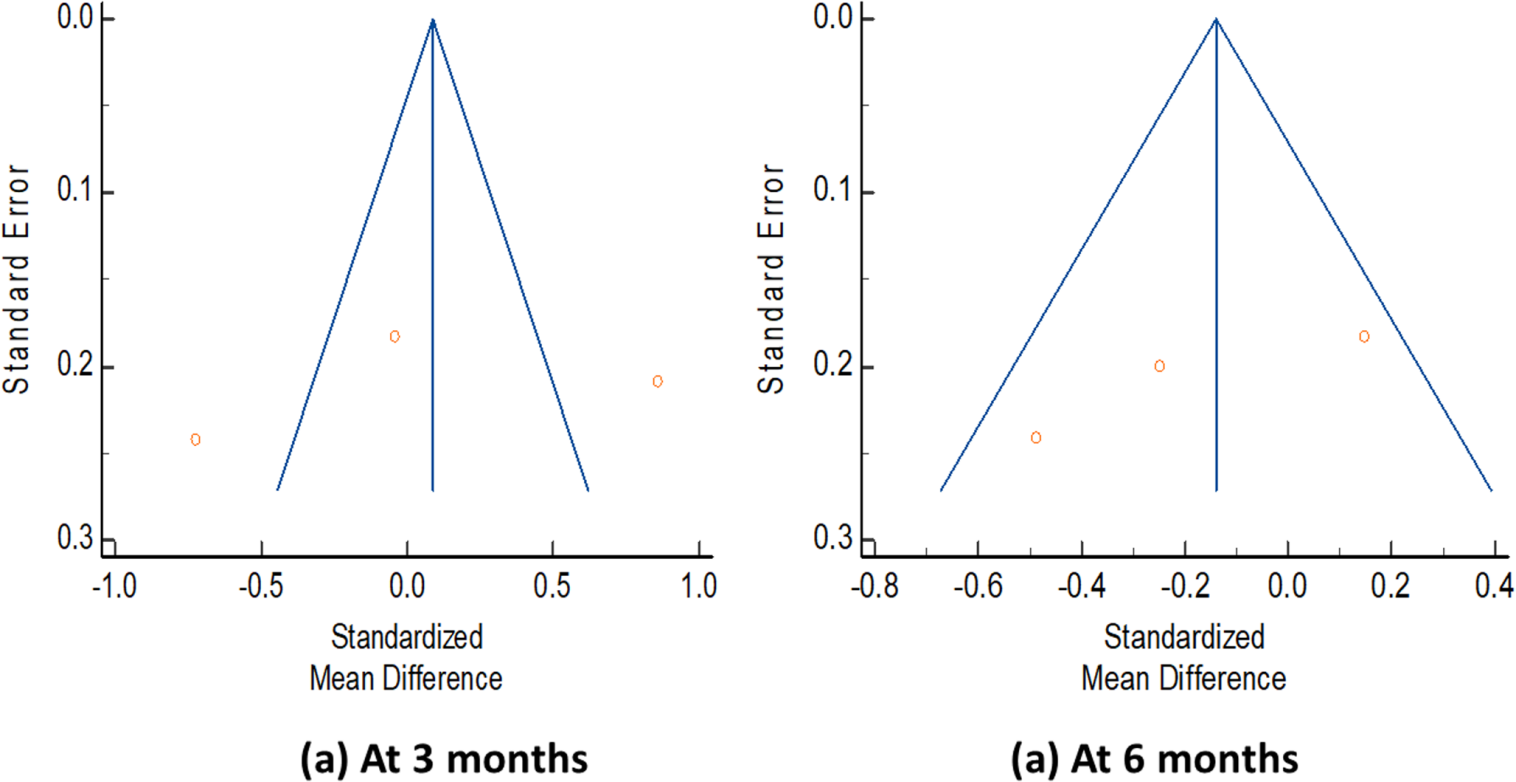
Publication bias for the change in postoperative disability (Oswestry Disability Index) from baseline to (**a**) 3 months postoperatively and (**b**) 6 months postoperatively after lumbar spine fusion surgery, comparing preoperative cognitive behavioral therapy with standard care

Change in the postoperative degree of disability from baseline to 3 months (SMD 0.04 [95% CI: − 0.81 to 0.88]; *p* = 0.93; I^2^ = 92%) and 6 months postoperatively (SMD − 0.17 [95% CI: − 0.53 to 0.19]; *p* = 0.36; I^2^ = 58%) was not significantly different between the perioperative CBT and control groups (Fig. 3). No publication bias was detected at 3 months (Egger’s test: Intercept = − 9.46, p-value = 0.75; Begg’s test: Kendall’s Tau = − 0.33, p-value = 0.6) and 6 months (Egger’s test: Intercept = − 10.39, p-value = 0.25; Begg’s test: Kendall’s Tau = − 1, p-value = 0.11) (Fig. 4).

### Secondary endpoint

**Fig. 5:**
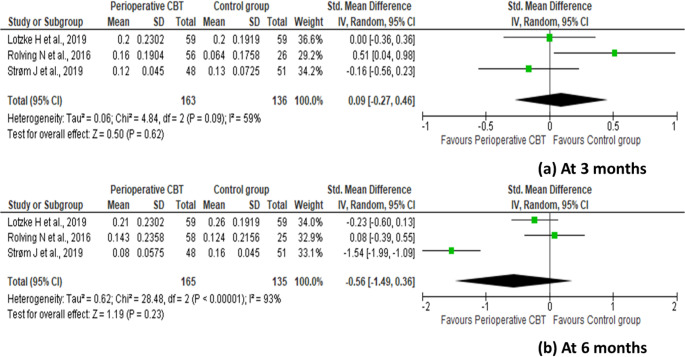
Forest plot with standardized mean difference (with 95% confidence intervals) for the change in the postoperative health-related quality of life (EQ-5D index) from baseline to (**a**) 3 months postoperatively and (**b**) 6 months postoperatively after lumbar spine fusion surgery, comparing preoperative cognitive behavioral therapy with standard care

**Fig. 6:**
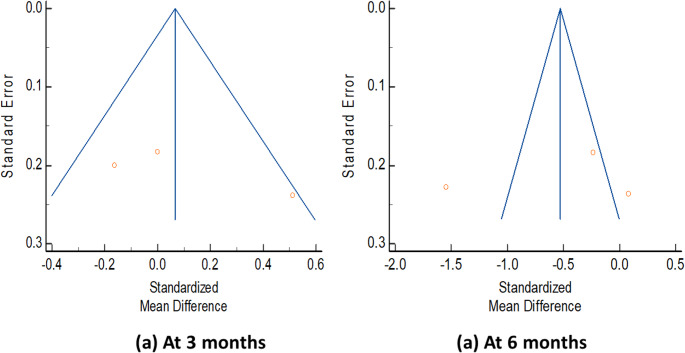
Publication bias for the change in postoperative health-related quality of life (EQ-5D index) from baseline to (**a**) 3 months postoperatively and (**b**) 6 months postoperatively after lumbar spine fusion surgery, comparing preoperative cognitive behavioral therapy with standard care

Change in postoperative health-related quality of life from baseline to 3 months (SMD 0.09 [95% CI: − 0.27 to 0.46]; *p* = 0.62; I^2^ = 59%) and 6 months after surgery (SMD − 0.56 [95% CI: − 1.49 to 0.36]; *p* = 0.23; I^2^ = 93%) was not significantly different statistically between the perioperative CBT and control groups (Fig. 5). No publication bias was detected for at 3 months (Egger’s test: Intercept = 9.76, p-value = 0.39; Begg’s test: Kendall’s Tau = 0.33, p-value = 0.6) or 6 months (Egger’s test: Intercept = − 7.09, p-value = 0.83; Begg’s test: Kendall’s Tau = 0.33, p-value = 0.6) (Fig. 6).

## Discussion

This systematic review examined the effectiveness of preoperative CBT for patients undergoing lumbar spine fusion surgery, analyzing three RCTs with 307 participants. Our meta-analysis revealed no statistically significant differences between CBT and control groups in either disability (ODI) or health-related quality of life (EQ-5D) at 3 or 6 months after surgery. Notably, substantial clinical and statistical heterogeneity was observed across studies, reflecting the diversity in CBT implementation approaches, which included group-based sessions, individualized therapy, and web-based platforms with varying intensity, duration, and delivery methods [22–24].

Our findings align with previous systematic reviews examining preoperative psychological interventions for spine surgery and other surgical populations, which have similarly demonstrated inconsistent effects [16, 17, 27–30]. Several factors may explain the lack of a significant effect in our meta-analysis. The “dosage” of CBT interventions *(ranging from four to six sessions)* may be insufficient to modify deeply entrenched psychological patterns in chronic pain patients approaching surgery [31]. The timing of outcome assessments (3 and 6 months) may be too early to capture the full psychological adaptation process following major surgery [32]. Moreover, the heterogeneity of the patient populations used in these studies may have obscured the potential benefits of CBT for specific subgroups, such as those with higher baseline catastrophizing or anxiety [33].

The included RCTs employed distinctly different implementation approaches, each with unique advantages and limitations. The group-based format utilized by Rolving N et al. offered peer support and cost-effectiveness but struggled with attendance compliance [24]. The individualized approach adopted by Lotzke H et al. provided personalized intervention but required intensive therapist resources [22]. Furthermore, the web-based platform implemented by Strøm J et al. demonstrated superior accessibility and consistent delivery but potentially reduced therapeutic alliance [23]. Adherence challenges were prevalent across all modalities, with work obligations, travel difficulties, and pain intensity cited as common barriers [22–26]. The most consistently implemented CBT components included pain neuroscience education, catastrophizing reduction, and activity pacing. Although these elements theoretically address key psychological barriers to surgical recovery [34], they may require more intensive application or better integration with physical rehabilitation to demonstrate significant effects [35].

While our meta-analysis showed no significant differences in disability or health-related quality of life measures, this may reflect limitations in our selected outcome measures rather than the inefficacy of CBT itself. The primary psychological targets of CBT, including catastrophizing, fear-avoidance beliefs, self-efficacy, and psychological distress, may improve without immediately translating into measurable changes in functional disability or general quality of life within the relatively short 6-month timeframe [12]. The mechanisms through which CBT might benefit spine surgery patients specifically include reducing preoperative anxiety that could amplify postoperative pain perception, establishing realistic recovery expectations, teaching active coping strategies for managing postoperative limitations, and improving self-efficacy for rehabilitation engagement: The psychological processes that may serve as important mediators between the intervention and more distal outcomes such as disability reduction [36, 37]. Additionally, the ODI and EQ-5D, while validated and widely used, may not capture the specific psychological dimensions most responsive to CBT intervention [38]. More sensitive, domain-specific measures such as the Pain Catastrophizing Scale, Tampa Scale for Kinesiophobia, and Pain Self-Efficacy Questionnaire might better detect meaningful changes resulting from psychological interventions, particularly in the early postoperative period when physical recovery is still ongoing [39].

Clinicians might consider more targeted approaches for patients with identified psychological risk factors [13]. Patients with preoperative anxiety and pain catastrophization may be most likely to benefit from such interventions [13]. Therefore, psychological risk stratification may be more effective than universal implementation, allowing for resource-efficient allocation of psychological interventions to those most likely to benefit. The integration of CBT principles within comprehensive prehabilitation programs that address physical and psychological readiness simultaneously would be a better approach to assess its potential [36]. Moreover, the extension of psychological support into the postoperative period when patients face the challenges of recovery and rehabilitation would provide a holistic psychological cover [40]. The heterogeneity of implementation approaches suggests that a standardized, evidence-based protocol specifically designed for patients scheduled to be managed with lumbar spine fusion surgery has yet to emerge.

Future research should therefore prioritize larger, adequately powered RCTs with stratification by psychological risk profiles to identify which patients benefit most from preoperative CBT. We would suggest stratification of patients based on the presence of primarily axial (discogenic or annular tear) pain versus that arising due to spinal instability, and hypothesize that the former group might have a higher likelihood of benefitting from preoperative CBT. Standardized, manualized interventions with clearly defined “active ingredients” would facilitate replication and implementation. Extended follow-up periods up to and beyond 24 months would better capture the long-term trajectory of psychological adaptation and functional recovery. Additionally, mixed-methods approaches incorporating qualitative patient experiences could illuminate the mechanisms through which psychological interventions influence the surgical experience.

Furthermore, studies should also investigate whether preoperative CBT might facilitate post-traumatic growth following surgery, as a potential step to transform the surgical experience from merely restorative to genuinely enhancing for psychological well-being. Novel constructs such as psychological flexibility, meaning-making, and identity reconstruction following physical limitation should also be examined as potential mediators between CBT and quality of life outcomes in this patient population, while mixed-methods approaches incorporating qualitative patient interviews would provide potentially valuable insights into how psychological interventions influence subjective quality of life in ways that standardized measures might not be able to capture.

The strengths of this systematic review include its rigorous methodology following PRISMA guidelines, rigorous methodological quality assessment, comprehensive assessment of implementation approaches, and validated meta-analytical approach. However, several limitations warrant consideration. The small number of included RCTs (*n* = 3) limited the generalizability of findings. All included RCTs demonstrated a high risk of bias across several methodological quality domains. Furthermore, substantial heterogeneity in interventions complicated direct comparisons, and the focus on only two specific outcome measures may have overlooked other important benefits of preoperative CBT. While our aim was to examine preoperative CBT interventions, all included studies delivered perioperative CBT programs with both pre- and postoperative components, which may limit the generalizability of findings to interventions delivered solely in the preoperative period. In addition to this, while publication bias analyses were reassuring, the limited number of studies reduced the reliability of these tests. These limitations collectively suggest that our findings should be interpreted as preliminary evidence rather than definitive conclusions regarding the effectiveness of preoperative CBT in the context of patient-reported outcomes for patients scheduled to be managed with lumbar spine fusion surgery.

## Conclusion

Our systematic review and meta-analysis of RCTs found that preoperative CBT significantly improved neither disability nor quality of life outcomes at 3 and 6 months following lumbar spine fusion surgery, though marked heterogeneity in intervention approaches and high risk of bias across studies preclude definitive conclusions about efficacy. The absence of significant effects should be contextualized within the methodological limitations of the current evidence base rather than interpreted as proof that psychological factors are unimportant in surgical recovery. These findings suggest a need for more nuanced clinical application of targeted interventions for patients with quantifiably elevated psychological risk factors, with defined cut-off points, delivered within comprehensive prehabilitation programs that address both physical and psychological readiness for surgery. Patients with preoperative anxiety and pain catastrophization may be most likely to benefit from such interventions. Therefore, psychological risk stratification may be more effective than universal implementation, allowing for resource-efficient allocation of psychological interventions to those most likely to benefit. To advance this field, researchers must prioritize methodologically rigorous trials that employ standardized interventions with fidelity monitoring, stratify participants using validated domain-specific psychological screening tools, measure both process variables and functional outcomes, extend follow-up up to and beyond 24 months to capture the full recovery trajectory, and apply implementation science frameworks to determine how evidence-based psychological care can be feasibly integrated into existing surgical pathways in a manner that is both clinically effective and economically sustainable across diverse healthcare settings for patients scheduled to be managed with lumbar spine fusion surgery.

## Supplementary Information

Below is the link to the electronic supplementary material.

Supplementary Material 1 (DOCX 220 KB)

## Data Availability

All data supporting the findings of this study are available within the paper and the related Information.
