# Supplementary material for: Effectiveness of preoperative cognitive behavioral therapy for patients undergoing lumbar spine fusion surgery: A systematic review focusing on patient-reported outcomes
Source: Neurosurg Rev. 2026 May 21;49(1):413. doi: 10.1007/s10143-026-04329-3 (PMC13190421; doi:10.1007/s10143-026-04329-3)
Supplement: Supplementary file 1 — Supplementary Material 1 (DOCX 220 KB) [file 10143_2026_4329_MOESM1_ESM.docx]

**Supplementary materials**

**Prospective Study Protocol & Search Strategy Implementation**

- **P**atients
  - Patients scheduled for lumbar spine fusion surgery, irrespective of etiology
    - **Exclusion:** Patients undergoing lumbar fusion as a secondary or adjunctive procedure rather than the primary intervention
- **I**ntervention
  - Preoperative Cognitive Behavioral Therapy (CBT) interventions delivered in any format (group-based, individual, or web-based)
    - **Exclusion:**
      - Psychological interventions lacking explicit cognitive-behavioral theoretical frameworks
      - Psychological interventions without standardized protocols
      - Exclusively postoperative psychological interventions
- **C**omparator
  - Standard preoperative care without structured psychological intervention
- **O**utcome
  - Primary outcome
    - Change in disability as measured by the Oswestry Disability Index (ODI) at 3 and 6 months after lumbar spine fusion surgery compared to baseline
  - Secondary outcome
  - Tertiary outcome
    - Change in health-related quality of life as measured by the EQ-5D index at 3 and 6 months after lumbar spine fusion surgery compared to baseline
  - Tertiary outcome
    - Implementation approaches of preoperative CBT for patients scheduled for lumbar spine fusion surgery
- **S**tudy design
  - **Inclusion:**
    - Randomized controlled trials
    - Prospective cohort studies
    - Retrospective cohort studies
    - Case-control studies
    - Case series with ≥ 5 participants
  - **Exclusion:**
    - Case series with < 5 participants
    - Case reports
    - Non-empirical publications (review articles, editorials, commentaries)
    - Conference abstracts without full-text availability
    - Non-English publications

MeSH terms were acquired for the aforementioned relevant terms from the MeSH resource of NCBI.

**Search strategy implemented on:** December 15, 2024

- **PubMed**
- "Lumbar Vertebrae"[MeSH Terms] OR "Lumbar Spine"[Title/Abstract] OR "Lumbar Region"[Title/Abstract] 87,259
- "Spinal Fusion"[MeSH Terms] OR "Spinal Arthrodesis"[Title/Abstract] OR "Lumbar Fusion"[Title/Abstract] OR "Lumbar Spinal Fusion"[Title/Abstract] 35,549
- "surgical procedures, operative"[MeSH Terms] OR "Surgical Treatment"[Title/Abstract] OR "Surgery"[Title/Abstract] 4,492,738
- #1 #2 #3 14,590
- "Cognitive Behavioral Therapy"[MeSH Terms] OR "Cognitive Behavioural Therapy"[Title/Abstract] OR "CBT"[Title/Abstract] OR "Cognitive Behavior Therapy"[Title/Abstract] OR "Cognitive Behaviour Therapy"[Title/Abstract]

49,682

- **#4 AND #5 150**
- **CSDR**
- MeSH descriptor: [Lumbar Vertebrae] explode all trees 3950
- (Lumbar):ti,ab,kw 22023
- #1 OR #2 22023
- MeSH descriptor: [Spinal Fusion] explode all trees 1665
- (Fusion):ti,ab,kw 10173
- #4 OR #5 10173
- MeSH descriptor: [Surgical Procedures, Operative] explode all trees 177464
- (surgical treatment):ti,ab,kw 68992
- (surgery):ti,ab,kw 302207
- #7 OR #8 OR #9 387020
- #3 AND #6 AND #10 2193
- MeSH descriptor: [Cognitive Behavioral Therapy] explode all trees 14628
- (Cognitive Behavioural Therapy):ti,ab,kw 27560
- (CBT):ti,ab,kw 12037
- (Cognitive Behavior Therapy):ti,ab,kw 13194
- (Cognitive Behaviour Therapy):ti,ab,kw 13194
- #12 OR #13 OR #14 OR #15 OR #16 35712
- **#11 AND #17 68**
- **Epistemonikos**
  - - (title:(lumbar) OR abstract:(lumbar)) 31,475
    - (title:(spine) OR abstract:(spine)) OR (title:(spinal) OR abstract:(spinal))

72,933

- - - (title:(fusion) OR abstract:(fusion)) 24,029
    - #1 AND #2 AND #3 3,033
    - (title:(Cognitive Behavioral Therapy) OR abstract:(Cognitive Behavioral Therapy)) 8,584
    - **#4 AND #5 7**
- Total articles obtained from search strategy implementation: 225
- Total articles obtained from manual search: 1
  - Duplicates removed: 45
    - Articles screened for inclusion: 181
      - **Articles included: 3**
- **Articles excluded (n= 178)**
  - **different research question (n= 152)**
    - Coe M, Mirza S, Sengupta D. The Role of Fusion for Discogenic Axial Back Pain Without Associated Leg Pain, Spondylolisthesis or Stenosis: An Evidence-Based Review. Semin Spine Surg. 2009;21(4):246-256. doi:10.1053/j.semss.2009.08.008
    - Bhandari M, Petrisor B, Busse JW, Drew B. Does lumbar surgery for chronic low-back pain make a difference?. CMAJ. 2005;173(4):365-366. doi:10.1503/cmaj.050884
    - Omair A, Holden M, Lie BA, Reikeras O, Brox JI. Treatment outcome of chronic low back pain and radiographic lumbar disc degeneration are associated with inflammatory and matrix degrading gene variants: a prospective genetic association study. BMC Musculoskelet Disord. 2013;14:105. Published 2013 Mar 22. doi:10.1186/1471-2474-14-105
    - Epstein D. Intensive rehabilitation may be more cost effective than surgical stabilization for chronic low back pain. Aust J Physiother. 2005;51(4):269. doi:10.1016/s0004-9514(05)70015-5
    - Nyström B. Lumbar Fusion or Non-Operative Care for Treatment of Presumed Discogenic Pain: A Randomized Study. clinicaltrials.gov; 2017. Accessed September 5, 2025. <https://clinicaltrials.gov/study/NCT02695576>
    - Mannion AF, Brox JI, Fairbank JC. Comparison of spinal fusion and nonoperative treatment in patients with chronic low back pain: long-term follow-up of three randomized controlled trials. Spine J. 2013;13(11):1438-1448. doi:10.1016/j.spinee.2013.06.101
    - Sveinsdottir V, Eriksen HR, Reme SE. Assessing the role of cognitive behavioral therapy in the management of chronic nonspecific back pain. J Pain Res. 2012;5:371-380. doi:10.2147/JPR.S25330
    - Abstracts for the BASS Meeting, Norwich 13–15 March 2013. Eur Spine J. 2013;22(1):54-78. doi:10.1007/s00586-013-2664-1
    - Chou R, Baisden J, Carragee EJ, Resnick DK, Shaffer WO, Loeser JD. Surgery for low back pain: a review of the evidence for an American Pain Society Clinical Practice Guideline. Spine (Phila Pa 1976). 2009;34(10):1094-1109. doi:10.1097/BRS.0b013e3181a105fc
    - Oral Presentations. Glob Spine J. 2023;13(2_suppl):4S-214S. doi:10.1177/21925682231166108
    - E-Posters. Glob Spine J. 2018;8(1_suppl):174S-374S. doi:10.1177/2192568218771072
    - ISRCTN21221956. Multidisciplinary programme for failed back surgery syndrome | Cochrane Library. doi:10.1002/central/CN-01972391
    - 43. The cortical trajectory fixation vs traditional pedicle screw fixation in the treatment of lumbar degenerative patients with osteoporosis: a prospective randomized controlled trail | Cochrane Library. doi:10.1002/central/CN-02611543
    - Lundberg M. Get Back, a Person-Centred Digital Program Targeting Physical Activity for Patients Undergoing Spinal Stenosis Surgery - a Randomized Feasibility Study. clinicaltrials.gov; 2024. Accessed September 5, 2025. <https://clinicaltrials.gov/study/NCT05806593>
    - Long-term (11-year) follow-up of three randomised controlled trials comparing spinal fusion and nonoperative treatment in patients with chronic low back pain | Cochrane Library. doi:10.1002/central/CN-01731120
    - 57. Resilience and self-efficacy are protective psychological factors for 12-month outcomes after lumbar spine surgery | Cochrane Library. doi:10.1002/central/CN-01978441
    - University Ghent. Cognitive Behavioural Therapy for Insomnia in Patients With Chronic Spinal Pain: A Multi-Center Randomized Controlled Trial. clinicaltrials.gov; 2022. Accessed September 6, 2025. <https://clinicaltrials.gov/study/NCT03482856>
    - Fujita R, Oda I, Tanaka H, et al. Real-world clinical accuracy of long cortical bone trajectory screw placement using a patient-specific template guide. J Spine Surg. 2024;10(3):468-478. doi:10.21037/jss-23-122
    - Yu Q, Zou ZL, Lu HG, Pan XK, Hu XQ, Shen ZH. Finite element analysis of biomechanical investigation on diverse internal fixation techniques in oblique lumbar interbody fusion. BMC Musculoskelet Disord. 2024;25(1):804. Published 2024 Oct 12. doi:10.1186/s12891-024-07887-z
    - Zeng W, Hu S, Zhu Z, Wang S, Guo L, Shi B. A 3D-CT Study of the Cortical Bone Trajectory Screw Placement Parameters Based on Lumbar CT. Orthop Surg. 2024;16(11):2771-2780. doi:10.1111/os.14202
    - Tao EX, Zhang RJ, Zhang B, Wang JQ, Zhou LP, Shen CL. Biomechanical changes of oblique lumbar interbody fusion with different fixation techniques in degenerative spondylolisthesis lumbar spine: a finite element analysis. BMC Musculoskelet Disord. 2024;25(1):664. Published 2024 Aug 24. doi:10.1186/s12891-024-07796-1
    - Matsukawa K, Kato T, Yanai Y, Fujiyoshi K, Yato Y. Influence of facetectomy, cross-link augmentation, and interbody procedure on progression of bone fusion in single-level posterior lumbar interbody fusion using the long cortical bone trajectory technique. J Neurosurg Spine. 2024;41(4):483-488. Published 2024 Aug 2. doi:10.3171/2024.5.SPINE231366
    - Shang Q, Luan H, Peng C, Song X. Comparative effectiveness of cortical bone trajectory screws and pedicle screws in the treatment of adjacent segment degeneration after lumbar fusion surgery: a systematic review and meta-analysis. J Orthop Surg Res. 2024;19(1):380. Published 2024 Jun 28. doi:10.1186/s13018-024-04865-y
    - Daher M, Nassar JE, Ikwuazom CP, et al. Cortical Trajectory versus Traditional Pedicle Screw Trajectory in Open Transforaminal Lumbar Interbody Fusion: Meta-Analysis of Complications and Clinical Outcomes. World Neurosurg. 2024;189:212-219. doi:10.1016/j.wneu.2024.06.062
    - Wang JQ, Zhang RJ, Zhou LP, et al. Design and radiological confirmation of 3-column cortical bone trajectory in the lumbar spine. J Neurosurg Spine. 2024;41(2):199-208. Published 2024 May 24. doi:10.3171/2024.2.SPINE231208
    - Zheng J, Wu Y, Guo C, Fang X, Ding T. Efficacy and Safety of Cortical Bone Trajectory Screws versus Pedicle Screws in Lumbar Fusion: A Systematic Review and Meta-Analysis. World Neurosurg. 2024;188:e233-e246. doi:10.1016/j.wneu.2024.05.090
    - Werthmann NJ 3rd, Gum JL, Nagata K, et al. Comparison of No Tap (two-step) and tapping robotic assisted cortical bone trajectory screw insertion. J Robot Surg. 2024;18(1):204. Published 2024 May 8. doi:10.1007/s11701-024-01890-1
    - Miyashita T, Kato K, Yunde A, Ataka H, Tanno T. Comparison of facet fusion rates and clinical outcomes between cortical bone trajectory screw and percutaneous pedicle screw fixation for degenerative lumbar spondylolisthesis. Spine J. 2024;24(7):1202-1210. doi:10.1016/j.spinee.2024.02.017
    - Zhu C, Liang J, Pan H, Zhang W. Biportal endoscopic-assisted cortical bone trajectory screw placement and lumbar interbody fusion. Acta Neurochir (Wien). 2024;166(1):74. Published 2024 Feb 8. doi:10.1007/s00701-024-05986-3
    - Nagata K, Gum JL, Brown M, et al. Risk Factors for Medial Breach During Robotic-Assisted Cortical Bone Trajectory Screw Insertion. World Neurosurg. 2024;184:e546-e553. doi:10.1016/j.wneu.2024.01.168
    - Liu C, Zhao M, Zhang W, et al. Biomechanical assessment of different transforaminal lumbar interbody fusion constructs in normal and osteoporotic condition: a finite element analysis. Spine J. 2024;24(6):1121-1131. doi:10.1016/j.spinee.2024.01.017
    - Wu C, Hu X, Liu R, et al. Comparison of the clinical and radiographic outcomes of cortical bone trajectory and traditional trajectory pedicle screw fixation in transforaminal lumbar interbody fusion: a randomized controlled trial. Eur Spine J. 2024;33(3):1069-1080. doi:10.1007/s00586-023-08086-5
    - Tuoheti A, Xiao Y, Wang Y, et al. Biomechanical evaluation of modified and traditional cortical bone trajectory technique on adjacent segment degeneration in transforaminal lumbar interbody fusion-finite element analysis. BMC Musculoskelet Disord. 2024;25(1):7. Published 2024 Jan 2. doi:10.1186/s12891-023-07103-4
    - Yamagishi A, Ishii M, Sakaura H, et al. The Influence of Titanium-coated Poryetheretherketone Cages in Fusion Status after Posterior Lumbar Interbody Fusion with Cortical Bone Trajectory Screw Fixation. World Neurosurg. 2024;183:e201-e209. doi:10.1016/j.wneu.2023.12.056
    - Zhang XN, Su QJ, Pei BQ, et al. The accuracy of cortical bone trajectory screw placement guided by spinous process clamp hardware in lumbar spinal surgery: a retrospective study. Sci Rep. 2023;13(1):16447. Published 2023 Sep 30. doi:10.1038/s41598-023-43406-1
    - Nagata K, Glassman SD, Brown ME, et al. Risk Factors of Screw Malposition in Robot-Assisted Cortical Bone Trajectory: Analysis of 1344 Consecutive Screws in 256 Patients. Spine (Phila Pa 1976). 2024;49(11):780-787. doi:10.1097/BRS.0000000000004827
    - Huang Y, Maimaiti A, Tian Y, Li Z, Kahaer A, Rexiti P. Biomechanical investigation of the hybrid lumbar fixation technique with traditional and cortical bone trajectories in transforaminal lumbar interbody fusion: finite element analysis. J Orthop Surg Res. 2023;18(1):549. Published 2023 Jul 31. doi:10.1186/s13018-023-04027-6
    - Liu DS, Wang YX, Rexiti P. Zhonghua Wai Ke Za Zhi. 2023;61(8):722-727. doi:10.3760/cma.j.cn112139-20230220-00074
    - Kong X, Li B, Xu L, et al. Safety and Efficacy of Cortical Bone Trajectory Screw Fixation Combined with Facet Fusion for the Treatment of Lumbar Degenerative Disease. Orthop Surg. 2023;15(6):1617-1626. doi:10.1111/os.13752
    - Schleifenbaum S, Vogl AC, Heilmann R, von der Hoeh NH, Heyde CE, Jarvers JS. Biomechanical comparative study of midline cortical vs. traditional pedicle screw trajectory in osteoporotic bone. BMC Musculoskelet Disord. 2023;24(1):395. Published 2023 May 18. doi:10.1186/s12891-023-06502-x
    - Fan K, Zhang D, Xue R, et al. Biomechanical Analysis of Double-Level Oblique Lumbar Fusion with Different Types of Fixation: A Finite Element-Based Study. Orthop Surg. 2023;15(5):1357-1365. doi:10.1111/os.13703
    - Lin Y, Xu J, Zheng W. The Fusion Rate of Cortical Bone Trajectory Screw Fixation and Pedicle Screw Fixations in L4-5 Interbody Fusion: A Retrospective Cohort Study. Orthop Surg. 2023;15(5):1281-1288. doi:10.1111/os.13704
    - Tang YX, Peng SL, Chen YW, Huang HM, Shih CT. Evaluating the contact anatomy and contact bone volume of spinal screws using a novel drilled surface image. PLoS One. 2023;18(4):e0282737. Published 2023 Apr 10. doi:10.1371/journal.pone.0282737
    - Kwon JW, Park Y, Lee BH, et al. A Comparison Between Cortical Bone Trajectory Screws And Traditional Pedicle Screws in Patients With Single-Level Lumbar Degenerative Spondylolisthesis: Five-Year Results. Spine (Phila Pa 1976). 2023;48(22):1617-1625. doi:10.1097/BRS.0000000000004523
    - Liu D, Kahaer A, Wang Y, et al. Comparison of CT values in traditional trajectory, traditional cortical bone trajectory, and modified cortical bone trajectory. BMC Surg. 2022;22(1):441. Published 2022 Dec 27. doi:10.1186/s12893-022-01893-5
    - Zhang XN, Zhou LJ, Su QJ, et al. Accuracy of cortical bone trajectory screw fixation guided by spinous process clamp guide in lumbosacral vertebrae: A cadaver study. Int J Med Robot. 2023;19(2):e2484. doi:10.1002/rcs.2484
    - Di Perna G, Marengo N, Matsukawa K, et al. Three-Dimensional Patient-Matched Template Guides Are Able to Increase Mean Diameter and Length and to Improve Accuracy of Cortical Bone Trajectory Screws: A 5-Year International Experience. World Neurosurg. 2023;170:e542-e549. doi:10.1016/j.wneu.2022.11.066
    - Zhang XN, Zhang YQ, Liu YZ, et al. Cortical screw placement with a spinous process clamp guide: a cadaver study accessing accuracy. BMC Surg. 2022;22(1):384. Published 2022 Nov 8. doi:10.1186/s12893-022-01829-z
    - Wu J, Yang D, Han Y, et al. Application of dual-trajectory screws in revision surgery for lumbar adjacent segment disease: a finite element study. J Orthop Surg Res. 2022;17(1):427. Published 2022 Sep 24. doi:10.1186/s13018-022-03317-9
    - Zhang P, Ye J, Huang L, et al. Comparison of Affected-Vertebra Fixation of Cortical Bone Trajectory Screw and Pedicle Screw for Lumbar Tuberculosis: A Minimum 3-Year Follow-Up. Biomed Res Int. 2022;2022:6312994. Published 2022 Jul 21. doi:10.1155/2022/6312994
    - Zhang HQ, Wang CC, Zhang RJ, et al. Predictors of accurate intrapedicular screw placement in single-level lumbar (L4-5) fusion: robot-assisted pedicle screw, traditional pedicle screw, and cortical bone trajectory screw insertion. BMC Surg. 2022;22(1):284. Published 2022 Jul 24. doi:10.1186/s12893-022-01733-6
    - Chung TT, Chu CL, Hueng DY, Lin SC. A parametric investigation on traditional and cortical bone trajectory screws for transpedicular fixation. BMC Musculoskelet Disord. 2022;23(1):612. Published 2022 Jun 27. doi:10.1186/s12891-022-05477-5
    - Han B, Ding H, Hai Y, et al. May the midline lumbar interbody fusion (MIDLIF) prevent the early radiographic adjacent segment degeneration? A minimum 3-year follow-up comparative study of MIDLIF in L4/5 with cortical bone trajectory screw versus traditional pedicle screw fixation. BMC Musculoskelet Disord. 2022;23(1):480. Published 2022 May 20. doi:10.1186/s12891-022-05363-0
    - Qiu L, Niu F, Wu Z, et al. Comparative Outcomes of Cortical Bone Trajectory Screw Fixation and Traditional Pedicle Screws in Lumbar Fusion: A Meta-Analysis. World Neurosurg. 2022;164:e436-e445. doi:10.1016/j.wneu.2022.04.129
    - Kahaer A, Zhou Z, Maitirouzi J, et al. Biomechanical Investigation of the Posterior Pedicle Screw Fixation System at Level L4-L5 Lumbar Segment with Traditional and Cortical Trajectories: A Finite Element Study. J Healthc Eng. 2022;2022:4826507. Published 2022 Mar 28. doi:10.1155/2022/4826507
    - Li Y, Chen L, Liu Y, et al. Accuracy and safety of robot-assisted cortical bone trajectory screw placement: a comparison of robot-assisted technique with fluoroscopy-assisted approach. BMC Musculoskelet Disord. 2022;23(1):328. Published 2022 Apr 6. doi:10.1186/s12891-022-05206-y
    - Zhang S, Liu Z, Lu C, et al. Oblique lateral interbody fusion combined with different internal fixations for the treatment of degenerative lumbar spine disease: a finite element analysis. BMC Musculoskelet Disord. 2022;23(1):206. Published 2022 Mar 4. doi:10.1186/s12891-022-05150-x
    - Ding H, Hai Y, Liu Y, et al. Cortical Trajectory Fixation Versus Traditional Pedicle-Screw Fixation in the Treatment of Lumbar Degenerative Patients with Osteoporosis: A Prospective Randomized Controlled Trial. Clin Interv Aging. 2022;17:175-184. Published 2022 Feb 23. doi:10.2147/CIA.S349533
    - Arzoglou V, Vial I, Hussain M, et al. Lumbar Fixation Using the Cortical Bone Trajectory Fixation: A Single Surgeon's Experience With 3-Year Follow-up. Oper Neurosurg. 2022;22(3):87-100. doi:10.1227/ONS.0000000000000042
    - Li Y, Chen Y, Liu Y, et al. Changes in Paraspinal Muscles and Facet Joints after Minimally Invasive Posterior Lumbar Interbody Fusion Using the Cortical Bone Trajectory Technique: A Prospective Study. Pain Res Manag. 2022;2022:2690291. Published 2022 Jan 12. doi:10.1155/2022/2690291
    - Kim KT, Song MG, Lee EC, Seo MS, Lee DY, Kim DH. Can the cortical bone trajectory screw technique be an alternative method to the pedicle screw in posterior lumbar fusion? A systematic review and metaanalysis. Acta Orthop Traumatol Turc. 2021;55(6):552-562. doi:10.5152/j.aott.2021.21169
    - Kuo YH, Kuo CH, Chang HK, et al. Cortical Bone Trajectory-Based Dynamic Stabilization. World Neurosurg. 2022;159:e416-e424. doi:10.1016/j.wneu.2021.12.061
    - Li J, Chen YL, Chen BH, et al. Zhonghua Yi Xue Za Zhi. 2021;101(45):3724-3729. doi:10.3760/cma.j.cn112137-20210416-00919
    - Shen CL, Zhang RJ. Zhonghua Yi Xue Za Zhi. 2021;101(45):3695-3699. doi:10.3760/cma.j.cn112137-20210416-00921
    - Sakaura H, Ikegami D, Fujimori T, Sugiura T, Yamada S, Mukai Y. Surgical outcomes after posterior lumbar interbody fusion using traditional trajectory screw fixation or cortical bone trajectory screw fixation: A comparative study between the polyetheretherketone cage and the same shape titanium-coated polyetheretherketone cage. Clin Neurol Neurosurg. 2021;209:106945. doi:10.1016/j.clineuro.2021.106945
    - Ishii M, Ohnishi A, Yamagishi A, Ohwada T. Freehand screw insertion technique without image guidance for the cortical bone trajectory screw in posterior lumbar interbody fusion: what affects screw misplacement?. J Neurosurg Spine. 2021;36(1):1-7. Published 2021 Sep 3. doi:10.3171/2021.2.SPINE202145
    - Sakaura H, Ikegami D, Fujimori T, Sugiura T, Mukai Y. Early Fusion Status After Posterior Lumbar Interbody Fusion With Cortical Bone Trajectory Screw Fixation or Traditional Trajectory Screw Fixation: A Comparison Between the Titanium-coated Polyetheretherketone Cage and the Same Shape Polyetheretherketone Cage. Clin Spine Surg. 2022;35(1):E47-E52. doi:10.1097/BSD.0000000000001237
    - He K, Dong C, Wei H, et al. A Minimally Invasive Technique Using Cortical Bone Trajectory Screws Assisted by 3D-Printed Navigation Templates in Lumbar Adjacent Segment Degeneration. Clin Interv Aging. 2021;16:1403-1413. Published 2021 Jul 20. doi:10.2147/CIA.S318525
    - Rosinski AA, Mittal A, Odeh K, et al. Alternatives to Traditional Pedicle Screws for Posterior Fixation of the Degenerative Lumbar Spine. JBJS Rev. 2021;9(7):e20.00177. Published 2021 Jul 28. doi:10.2106/JBJS.RVW.20.00177
    - Kumar KK, Parikh B, Jabarkheel R, Dirlikov B, Singh H. Fluoroscopic versus CT-guided cortical bone trajectory pedicle screw fixation: Comparing trajectory related complications. J Clin Neurosci. 2021;89:354-359. doi:10.1016/j.jocn.2021.05.048
    - Spirig JM, Winkler E, Cornaz F, et al. Biomechanical performance of bicortical versus pericortical bone trajectory (CBT) pedicle screws. Eur Spine J. 2021;30(8):2292-2300. doi:10.1007/s00586-021-06878-1
    - Kim KT, Song MG, Park YJ, Lee DY, Kim DH. Cortical Trajectory Screw Fixation in Lumbar Spine Surgery: A Review of the Existing Literature. Asian Spine J. 2022;16(1):127-140. doi:10.31616/asj.2020.0575
    - Zhang RJ, Zhou LP, Zhang L, et al. The Rates and Risk Factors of Intra-Pedicular Accuracy and Proximal Facet Joint Violation for Single-Level Degenerative Lumbar Diseases: Cortical Bone Trajectory Versus Traditional Trajectory Pedicle Screw. Spine (Phila Pa 1976). 2021;46(23):E1274-E1282. doi:10.1097/BRS.0000000000004083
    - Zhang L, Li HM, Zhang R, Zhang H, Shen CL. Biomechanical Changes of Adjacent and Fixed Segments Through Cortical Bone Trajectory Screw Fixation versus Traditional Trajectory Screw Fixation in the Lumbar Spine: A Finite Element Analysis. World Neurosurg. 2021;151:e447-e456. doi:10.1016/j.wneu.2021.04.061
    - Terai H, Tamai K, Takahashi S, et al. Clinical Comparison of Combined Cortical Bone Trajectory and Transarticular Surface Screw Versus Standard Pedicle Screw Insertion by Wiltse Approach for L5 Isthmic Spondylolisthesis. Clin Spine Surg. 2021;34(10):E580-E587. doi:10.1097/BSD.0000000000001170
    - Zhao Y, Liang J, Luo H, Xu Y, Lu S. Double-trajectory lumbar screw placement guided by a set of 3D-printed surgical guide templates: a cadaver study. BMC Musculoskelet Disord. 2021;22(1):296. Published 2021 Mar 22. doi:10.1186/s12891-021-04149-0
    - Ding H, Han B, Hai Y, et al. The Feasibility of Assessing the Cortical Bone Trajectory Screw Placement Accuracy Using a Traditional Pedicle Screw Insertion Evaluation System. Clin Spine Surg. 2021;34(2):E112-E120. doi:10.1097/BSD.0000000000001059
    - Delgado-Fernández J, Frade-Porto N, Blasco G, et al. Simulation with 3D Neuronavigation for Learning Cortical Bone Trajectory Screw Placement. J Neurol Surg A Cent Eur Neurosurg. 2021;82(3):262-269. doi:10.1055/s-0040-1715485
    - Ding HT, Hai Y, Liu YZ, et al. Zhonghua Yi Xue Za Zhi. 2020;100(43):3437-3442. doi:10.3760/cma.j.cn112137-20200417-01218
    - Chen CH, Chen DC, Huang HM, et al. Level-based analysis of screw loosening with cortical bone trajectory screws in patients with lumbar degenerative disease. Medicine (Baltimore). 2020;99(40):e22186. doi:10.1097/MD.0000000000022186
    - Lee CK, Kim D, An SB, et al. An optimal cortical bone trajectory technique to prevent early surgical complications. Br J Neurosurg. 2024;38(2):208-214. doi:10.1080/02688697.2020.1821172
    - Kotani Y, Ikeura A, Tokunaga H, Saito T. Single-level controlled comparison of OLIF51 and percutaneous screw in lateral position versus MIS-TLIF for lumbosacral degenerative disorders: Clinical and radiologic study. J Orthop Sci. 2021;26(5):756-764. doi:10.1016/j.jos.2020.08.005
    - Chen H, Liu S, Zhang J, et al. Zhongguo Xiu Fu Chong Jian Wai Ke Za Zhi. 2020;34(9):1142-1148. doi:10.7507/1002-1892.202001070
    - Chang CC, Kuo CH, Chang HK, et al. Minimally invasive dynamic screw stabilization using cortical bone trajectory. BMC Musculoskelet Disord. 2020;21(1):605. Published 2020 Sep 10. doi:10.1186/s12891-020-03629-z
    - Zhang L, Tian N, Yang J, Ni W, Jin L. Risk of pedicle and spinous process violation during cortical bone trajectory screw placement in the lumbar spine. BMC Musculoskelet Disord. 2020;21(1):536. Published 2020 Aug 11. doi:10.1186/s12891-020-03535-4
    - Lai Z, Shi SY, Fei J, Han GH, Hu SP. Zhongguo Gu Shang. 2020;33(7):636-642. doi:10.12200/j.issn.1003-0034.2020.07.009
    - Liu CW, Wang LL, Xu YK, et al. Traditional and cortical trajectory screws of static and dynamic lumbar fixation- a finite element study. BMC Musculoskelet Disord. 2020;21(1):463. Published 2020 Jul 14. doi:10.1186/s12891-020-03437-5
    - Maruo K, Arizumi F, Kusuyama K, Kishima K, Tachibana T. Accuracy and safety of cortical bone trajectory screw placement by an inexperienced surgeon using 3D patient-specific guides for transforaminal lumbar interbody fusion. J Clin Neurosci. 2020;78:147-152. doi:10.1016/j.jocn.2020.04.090
    - Petrone S, Marengo N, Ajello M, et al. Cortical bone trajectory technique's outcomes and procedures for posterior lumbar fusion: A retrospective study. J Clin Neurosci. 2020;76:25-30. doi:10.1016/j.jocn.2020.04.070
    - DE Bonis P, Chiccoli M, Visani J, Cavallo MA, Scerrati A. Functional outcome of patients with unstable single-level/two-level lumbar stenosis treated with decompression plus divergent screws (cortical bone trajectory) or percutaneous convergent pedicle screws. J Neurosurg Sci. 2022;66(6):576-581. doi:10.23736/S0390-5616.20.04893-6
    - Huang Z, Sun N, Ren J, et al. Zhongguo Xiu Fu Chong Jian Wai Ke Za Zhi. 2020;34(2):162-167. doi:10.7507/1002-1892.201908087
    - Le XF, Shi Z, Wang QL, Xu YF, Zhao JW, Tian W. Rate and Risk Factors of Superior Facet Joint Violation during Cortical Bone Trajectory Screw Placement: A Comparison of Robot-Assisted Approach with a Conventional Technique. Orthop Surg. 2020;12(1):133-140. doi:10.1111/os.12598
    - Le X, Shi Z, Xu Y, Wang Q, Zhao J, Tian W. Incidence and Risk Factors of Superior Facet Joint Violation in Percutaneous and Open Instrumentation Using Cortical Bone Trajectory Technique: A Comparison of Different Techniques. Clin Spine Surg. 2020;33(3):E127-E134. doi:10.1097/BSD.0000000000000917
    - Sakaura H, Ikegami D, Fujimori T, et al. Early cephalad adjacent segment degeneration after posterior lumbar interbody fusion: a comparative study between cortical bone trajectory screw fixation and traditional trajectory screw fixation. J Neurosurg Spine. 2019;32(2):155-159. Published 2019 Oct 18. doi:10.3171/2019.8.SPINE19631
    - Miyashita T, Kato K, Takaoka H, Ataka H, Tanno T. A simple formula for predicting diameter of safely inserted cortical bone trajectory screws for fixation of the lower lumbar spine. J Orthop Sci. 2019;24(6):974-978. doi:10.1016/j.jos.2019.08.019
    - Hu JN, Yang XF, Li CM, Li XX, Ding YZ. Comparison of cortical bone trajectory versus pedicle screw techniques in lumbar fusion surgery: A meta-analysis. Medicine (Baltimore). 2019;98(33):e16751. doi:10.1097/MD.0000000000016751
    - Marengo N, Matsukawa K, Monticelli M, et al. Cortical Bone Trajectory Screw Placement Accuracy with a Patient-Matched 3-Dimensional Printed Guide in Lumbar Spinal Surgery: A Clinical Study. World Neurosurg. 2019;130:e98-e104. doi:10.1016/j.wneu.2019.05.241
    - Matsukawa K, Kaito T, Abe Y. Accuracy of cortical bone trajectory screw placement using patient-specific template guide system. Neurosurg Rev. 2020;43(4):1135-1142. doi:10.1007/s10143-019-01140-1
    - Liu L, Zhang S, Liu G, Yang B, Wu X. Early Clinical Outcome of Lumbar Spinal Fixation With Cortical Bone Trajectory Pedicle Screws in Patients With Osteoporosis With Degenerative Disease. Orthopedics. 2019;42(5):e465-e471. doi:10.3928/01477447-20190604-01
    - Liu YZ, Hai Y, Zhang XN, et al. Zhonghua Yi Xue Za Zhi. 2019;99(19):1473-1478. doi:10.3760/cma.j.issn.0376-2491.2019.19.008
    - Wang J, He X, Sun T. Comparative clinical efficacy and safety of cortical bone trajectory screw fixation and traditional pedicle screw fixation in posterior lumbar fusion: a systematic review and meta-analysis. Eur Spine J. 2019;28(7):1678-1689. doi:10.1007/s00586-019-05999-y
    - Penner F, Marengo N, Ajello M, et al. Preoperative 3D CT Planning for Cortical Bone Trajectory Screws: A Retrospective Radiological Cohort Study. World Neurosurg. 2019;126:e1468-e1474. doi:10.1016/j.wneu.2019.03.121
    - Dayani F, Chen YR, Johnson E, et al. Minimally invasive lumbar pedicle screw fixation using cortical bone trajectory - Screw accuracy, complications, and learning curve in 100 screw placements. J Clin Neurosci. 2019;61:106-111. doi:10.1016/j.jocn.2018.10.131
    - Huang HM, Chen CH, Lee HC, et al. Minimal invasive surgical technique in midline lumbar inter-body fusion: A technique note. J Clin Neurosci. 2018;55:103-108. doi:10.1016/j.jocn.2018.06.033
    - Hayashi K, Toyoda H, Terai H, et al. Comparison of minimally invasive decompression and combined minimally invasive decompression and fusion in patients with degenerative spondylolisthesis with instability. J Clin Neurosci. 2018;57:79-85. doi:10.1016/j.jocn.2018.08.032
    - Shi S, Ying X, Zheng Q, et al. Application of Cortical Bone Trajectory Screws in Elderly Patients with Lumbar Spinal Tuberculosis. World Neurosurg. 2018;117:e82-e89. doi:10.1016/j.wneu.2018.05.168
    - Gao H, Zhang R, Jia C, et al. Novel Placement of Cortical Bone Trajectory Screws in the Lumbar Spine: A Radiographic and Cadaveric Study. Clin Spine Surg. 2018;31(6):E329-E336. doi:10.1097/BSD.0000000000000651
    - Asamoto S, Kojima K, Winking M, et al. Optimized Screw Trajectory for Lumbar Cortical Bone Trajectory Pedicle Screws Based on Clinical Outcome: Evidence Favoring the Buttress Effect Theory. J Neurol Surg A Cent Eur Neurosurg. 2018;79(6):464-470. doi:10.1055/s-0038-1641147
    - Hussain I, Virk MS, Link TW, Tsiouris AJ, Elowitz E. Posterior Lumbar Interbody Fusion with 3D-Navigation Guided Cortical Bone Trajectory Screws for L4/5 Degenerative Spondylolisthesis: 1-Year Clinical and Radiographic Outcomes. World Neurosurg. 2018;110:e504-e513. doi:10.1016/j.wneu.2017.11.034
    - Feng Z, Li X, Tang Q, et al. Transforaminal lumbar interbody fusion with cortical bone trajectory screws versus traditional pedicle screws fixation: a study protocol of randomised controlled trial. BMJ Open. 2017;7(10):e017227. Published 2017 Oct 22. doi:10.1136/bmjopen-2017-017227
    - Senoglu M, Karadag A, Kinali B, Bozkurt B, Middlebrooks EH, Grande AW. Cortical Bone Trajectory Screw for Lumbar Fixation: A Quantitative Anatomic and Morphometric Evaluation. World Neurosurg. 2017;103:694-701. doi:10.1016/j.wneu.2017.03.137
    - Keorochana G, Pairuchvej S, Trathitephun W, Arirachakaran A, Predeeprompan P, Kongtharvonskul J. Comparative Outcomes of Cortical Screw Trajectory Fixation and Pedicle Screw Fixation in Lumbar Spinal Fusion: Systematic Review and Meta-analysis. World Neurosurg. 2017;102:340-349. doi:10.1016/j.wneu.2017.03.010
    - Madera M, Brady J, Deily S, et al. The role of physical therapy and rehabilitation after lumbar fusion surgery for degenerative disease: a systematic review. J Neurosurg Spine. 2017;26(6):694-704. doi:10.3171/2016.10.SPINE16627
    - Takenaka S, Mukai Y, Tateishi K, Hosono N, Fuji T, Kaito T. Clinical Outcomes After Posterior Lumbar Interbody Fusion: Comparison of Cortical Bone Trajectory and Conventional Pedicle Screw Insertion. Clin Spine Surg. 2017;30(10):E1411-E1418. doi:10.1097/BSD.0000000000000514
    - Sakaura H, Miwa T, Yamashita T, Kuroda Y, Ohwada T. Posterior lumbar interbody fusion with cortical bone trajectory screw fixation versus posterior lumbar interbody fusion using traditional pedicle screw fixation for degenerative lumbar spondylolisthesis: a comparative study. J Neurosurg Spine. 2016;25(5):591-595. doi:10.3171/2016.3.SPINE151525
    - Chin KR, Pencle FJR, Coombs AV, et al. Clinical Outcomes With Midline Cortical Bone Trajectory Pedicle Screws Versus Traditional Pedicle Screws in Moving Lumbar Fusions From Hospitals to Outpatient Surgery Centers. Clin Spine Surg. 2017;30(6):E791-E797. doi:10.1097/BSD.0000000000000436
    - Mullin JP, Perlmutter B, Schmidt E, Benzel E, Steinmetz MP. Radiographic feasibility study of cortical bone trajectory and traditional pedicle screw dual trajectories. J Neurosurg Spine. 2016;25(6):727-732. doi:10.3171/2016.4.SPINE151483
    - Dabbous B, Brown D, Tsitlakidis A, Arzoglou V. Clinical outcomes during the learning curve of MIDline Lumbar Fusion (MIDLF®) using the cortical bone trajectory. Acta Neurochir (Wien). 2016;158(7):1413-1420. doi:10.1007/s00701-016-2810-8
    - Hung CW, Wu MF, Hong RT, Weng MJ, Yu GF, Kao CH. Comparison of multifidus muscle atrophy after posterior lumbar interbody fusion with conventional and cortical bone trajectory. Clin Neurol Neurosurg. 2016;145:41-45. doi:10.1016/j.clineuro.2016.03.005
    - Orita S, Inage K, Kubota G, et al. One-Year Prospective Evaluation of the Technique of Percutaneous Cortical Bone Trajectory Spondylodesis in Comparison with Percutaneous Pedicle Screw Fixation: A Preliminary Report with Technical Note. J Neurol Surg A Cent Eur Neurosurg. 2016;77(6):531-537. doi:10.1055/s-0035-1566118
    - Matsukawa K, Kato T, Yato Y, et al. Incidence and Risk Factors of Adjacent Cranial Facet Joint Violation Following Pedicle Screw Insertion Using Cortical Bone Trajectory Technique. Spine (Phila Pa 1976). 2016;41(14):E851-E856. doi:10.1097/BRS.0000000000001459
    - Matsukawa K, Yato Y, Imabayashi H, Hosogane N, Asazuma T, Nemoto K. Biomechanical Evaluation of Cross Trajectory Technique for Pedicle Screw Insertion: Combined Use of Traditional Trajectory and Cortical Bone Trajectory. Orthop Surg. 2015;7(4):317-323. doi:10.1111/os.12212
    - Mai HT, Mitchell SM, Hashmi SZ, Jenkins TJ, Patel AA, Hsu WK. Differences in bone mineral density of fixation points between lumbar cortical and traditional pedicle screws. Spine J. 2016;16(7):835-841. doi:10.1016/j.spinee.2015.11.034
    - Greenwood J, McGregor A, Jones F, Mullane J, Hurley M. Rehabilitation Following Lumbar Fusion Surgery: A Systematic Review and Meta-Analysis. Spine (Phila Pa 1976). 2016;41(1):E28-E36. doi:10.1097/BRS.0000000000001132
    - Patel SS, Cheng WK, Danisa OA. Early complications after instrumentation of the lumbar spine using cortical bone trajectory technique. J Clin Neurosci. 2016;24:63-67. doi:10.1016/j.jocn.2015.07.018
    - Cheng WK, Akpolat YT, İnceoğlu S, Patel S, Danisa OA. Pars and pedicle fracture and screw loosening associated with cortical bone trajectory: a case series and proposed mechanism through a cadaveric study. Spine J. 2016;16(2):e59-e65. doi:10.1016/j.spinee.2015.09.046
    - Zhang H, Ajiboye RM, Shamie AN, Wu Q, Chen Q, Chen W. Morphometric measurement of the lumbosacral spine for minimally invasive cortical bone trajectory implant using computed tomography. Eur Spine J. 2016;25(3):870-876. doi:10.1007/s00586-015-4224-3
    - Ohkawa T, Iwatsuki K, Ohnishi Y, Ninomiya K, Yoshimine T. Isthmus-guided Cortical Bone Trajectory Reduces Postoperative Increases in Serum Creatinine Phosphokinase Concentrations. Orthop Surg. 2015;7(3):232-238. doi:10.1111/os.12189
    - Phan K, Hogan J, Maharaj M, Mobbs RJ. Cortical Bone Trajectory for Lumbar Pedicle Screw Placement: A Review of Published Reports. Orthop Surg. 2015;7(3):213-221. doi:10.1111/os.12185
    - Matsukawa K, Taguchi E, Yato Y, et al. Evaluation of the Fixation Strength of Pedicle Screws Using Cortical Bone Trajectory: What Is the Ideal Trajectory for Optimal Fixation?. Spine (Phila Pa 1976). 2015;40(15):E873-E878. doi:10.1097/BRS.0000000000000983
    - Greenwood J, McGregor A, Jones F, Hurley M. Evaluating rehabilitation following lumbar fusion surgery (REFS): study protocol for a randomised controlled trial. Trials. 2015;16:251. Published 2015 Jun 4. doi:10.1186/s13063-015-0751-9
    - Kojima K, Asamoto S, Kobayashi Y, Ishikawa M, Fukui Y. Cortical bone trajectory and traditional trajectory--a radiological evaluation of screw-bone contact. Acta Neurochir (Wien). 2015;157(7):1173-1178. doi:10.1007/s00701-015-2432-6
    - Song T, Hsu WK, Ye T. Lumbar pedicle cortical bone trajectory screw. Chin Med J (Engl). 2014;127(21):3808-3813.
    - Archer KR, Coronado RA, Haug CM, et al. A comparative effectiveness trial of postoperative management for lumbar spine surgery: changing behavior through physical therapy (CBPT) study protocol. BMC Musculoskelet Disord. 2014;15:325. Published 2014 Oct 1. doi:10.1186/1471-2474-15-325
    - Takata Y, Matsuura T, Higashino K, et al. Hybrid technique of cortical bone trajectory and pedicle screwing for minimally invasive spine reconstruction surgery: a technical note. J Med Invest. 2014;61(3-4):388-392. doi:10.2152/jmi.61.388
    - Iwatsuki K, Yoshimine T, Ohnishi Y, Ninomiya K, Ohkawa T. Isthmus-guided cortical bone trajectory for pedicle screw insertion. Orthop Surg. 2014;6(3):244-248. doi:10.1111/os.12122
    - Mizuno M, Kuraishi K, Umeda Y, Sano T, Tsuji M, Suzuki H. Midline lumbar fusion with cortical bone trajectory screw. Neurol Med Chir (Tokyo). 2014;54(9):716-721. doi:10.2176/[nmc.st](http://nmc.st).2013-0395
    - Matsukawa K, Yato Y, Kato T, Imabayashi H, Asazuma T, Nemoto K. Cortical bone trajectory for lumbosacral fixation: penetrating S-1 endplate screw technique: technical note. J Neurosurg Spine. 2014;21(2):203-209. doi:10.3171/2014.3.SPINE13665
    - Rodriguez A, Neal MT, Liu A, Somasundaram A, Hsu W, Branch CL Jr. Novel placement of cortical bone trajectory screws in previously instrumented pedicles for adjacent-segment lumbar disease using CT image-guided navigation. Neurosurg Focus. 2014;36(3):E9. doi:10.3171/2014.1.FOCUS13521
    - Matsukawa K, Yato Y, Kato T, Imabayashi H, Asazuma T, Nemoto K. In vivo analysis of insertional torque during pedicle screwing using cortical bone trajectory technique. Spine (Phila Pa 1976). 2014;39(4):E240-E245. doi:10.1097/BRS.0000000000000116
    - Mirza SK, Deyo RA, Heagerty PJ, Turner JA, Martin BI, Comstock BA. One-year outcomes of surgical versus nonsurgical treatments for discogenic back pain: a community-based prospective cohort study. Spine J. 2013;13(11):1421-1433. doi:10.1016/j.spinee.2013.05.047
    - Willems P. Decision making in surgical treatment of chronic low back pain: the performance of prognostic tests to select patients for lumbar spinal fusion. Acta Orthop Suppl. 2013;84(349):1-35. doi:10.3109/17453674.2012.753565
    - Froholdt A, Reikeraas O, Holm I, Keller A, Brox JI. No difference in 9-year outcome in CLBP patients randomized to lumbar fusion versus cognitive intervention and exercises. Eur Spine J. 2012;21(12):2531-2538. doi:10.1007/s00586-012-2382-0
    - Froholdt A, Holm I, Keller A, Gunderson RB, Reikeraas O, Brox JI. No difference in long-term trunk muscle strength, cross-sectional area, and density in patients with chronic low back pain 7 to 11 years after lumbar fusion versus cognitive intervention and exercises. Spine J. 2011;11(8):718-725. doi:10.1016/j.spinee.2011.06.004
    - Chang KW, Leng X, Zhao W, Chen YY, Chen TC, Chang KI. Broader curve criteria for selective thoracic fusion. Spine (Phila Pa 1976). 2011;36(20):1658-1664. doi:10.1097/BRS.0b013e318215fa73
    - Brox JI, Nygaard ØP, Holm I, Keller A, Ingebrigtsen T, Reikerås O. Four-year follow-up of surgical versus non-surgical therapy for chronic low back pain. Ann Rheum Dis. 2010;69(9):1643-1648. doi:10.1136/ard.2009.108902
    - Keller A, Brox JI, Reikerås O. Predictors of change in trunk muscle strength for patients with chronic low back pain randomized to lumbar fusion or cognitive intervention and exercises. Pain Med. 2008;9(6):680-687. doi:10.1111/j.1526-4637.2007.00333.x
    - Mirza SK, Deyo RA. Systematic review of randomized trials comparing lumbar fusion surgery to nonoperative care for treatment of chronic back pain. Spine (Phila Pa 1976). 2007;32(7):816-823. doi:10.1097/01.brs.0000259225.37454.38
    - Polomano RC, Marcotte PJ, Farrar JT. Spinal fusion or exercise and cognitive intervention? In search of the answers. Pain. 2006;122(1-2):4-5. doi:10.1016/j.pain.2006.01.028
    - Fairbank J, Frost H, Wilson-MacDonald J, et al. Randomised controlled trial to compare surgical stabilisation of the lumbar spine with an intensive rehabilitation programme for patients with chronic low back pain: the MRC spine stabilisation trial. BMJ. 2005;330(7502):1233. doi:10.1136/bmj.38441.620417.8F
    - Keller A, Brox JI, Gunderson R, Holm I, Friis A, Reikerås O. Trunk muscle strength, cross-sectional area, and density in patients with chronic low back pain randomized to lumbar fusion or cognitive intervention and exercises. Spine (Phila Pa 1976). 2004;29(1):3-8. doi:10.1097/[01.BRS.0000103946.26548.EB](http://01.brs.0000103946.26548.eb)
    - Brox JI, Sørensen R, Friis A, et al. Randomized clinical trial of lumbar instrumented fusion and cognitive intervention and exercises in patients with chronic low back pain and disc degeneration. Spine (Phila Pa 1976). 2003;28(17):1913-1921. doi:10.1097/01.BRS.0000083234.62751.7A
  - **Different intervention (n= 11)**
    - Tegner H, Esbensen BA, Henriksen M, et al. The effect of graded activity and pain education (GAPE): an early post-surgical rehabilitation programme after lumbar spinal fusion-study protocol for a randomized controlled trial. Trials. 2020;21(1):791. Published 2020 Sep 15. doi:10.1186/s13063-020-04719-y
    - Abbott AD, Tyni-Lenné R, Hedlund R. Early rehabilitation targeting cognition, behavior, and motor function after lumbar fusion: a randomized controlled trial. Spine (Phila Pa 1976). 2010;35(8):848-857. doi:10.1097/BRS.0b013e3181d1049f
    - Monticone M, Ferrante S, Teli M, et al. Management of catastrophising and kinesiophobia improves rehabilitation after fusion for lumbar spondylolisthesis and stenosis. A randomised controlled trial. Eur Spine J. 2014;23(1):87-95. doi:10.1007/s00586-013-2889-z
    - Tegner H. The Effect of Graded Activity and Pain Education (GAPE) for Patients Early After Lumbar Spinal Fusion. clinicaltrials.gov; 2024. Accessed September 6, 2025. <https://clinicaltrials.gov/study/NCT04103970>
    - Scarone P. The Impact of Cognitive Behavioral Therapy (CBT) on Treatment Outcome After Lumbar Spinal Fusion Surgery in Patients With High Pain Catastrophizing: A Two-Center Randomized Controlled Trial of CBT vs. Education Plus Usual Care. clinicaltrials.gov; 2020. Accessed September 6, 2025. <https://clinicaltrials.gov/study/NCT03969602>
    - Trajectories of PROMIS physical function, pain interference, and participation in social roles in 24 months following lumbar spine surgery | Cochrane Library. doi:10.1002/central/CN-02259758
    - Pennings JS, Coronado RA, Master H, Skolasky RL, Wegener S, Archer KR. P137. Trajectories of PROMIS physical function, pain interference, and spine legacy measures in 24 months following lumbar spine surgery. Spine J. 2021;21(9):S208. doi:10.1016/j.spinee.2021.05.345
    - Monticone M, Ambrosini E, Rocca B, Foti C, Ferrante S. Responsiveness and minimal clinically important changes for the Tampa Scale of Kinesiophobia after lumbar fusion during cognitive behavioral rehabilitation. Eur J Phys Rehabil Med. 2017;53(3):351-358. doi:10.23736/S1973-9087.16.04362-8
    - Lindgreen P, Rolving N, Nielsen CV, Lomborg K. Interdisciplinary Cognitive-Behavioral Therapy as Part of Lumbar Spinal Fusion Surgery Rehabilitation: Experience of Patients With Chronic Low Back Pain. Orthop Nurs. 2016;35(4):238-247. doi:10.1097/NOR.0000000000000259
    - Rolving N, Oestergaard LG, Willert MV, et al. Description and design considerations of a randomized clinical trial investigating the effect of a multidisciplinary cognitive-behavioural intervention for patients undergoing lumbar spinal fusion surgery. BMC Musculoskelet Disord. 2014;15:62. Published 2014 Mar 3. doi:10.1186/1471-2474-15-62
    - Brox JI, Reikerås O, Nygaard Ø, et al. Lumbar instrumented fusion compared with cognitive intervention and exercises in patients with chronic back pain after previous surgery for disc herniation: a prospective randomized controlled study. Pain. 2006;122(1-2):145-155. doi:10.1016/j.pain.2006.01.027
  - **Conference abstract (n= 3)**
    - Does a preoperative cognitive-behavioural intervention affect postsurgical pain, mobilisation and length of hospitalisation in lumbar spinal fusion patients? | Cochrane Library. doi:10.1002/central/CN-01063547
    - Does a web-based spine platform featuring social interaction and animated information affect patient reported outcomes in patients undertaking lumbar spine fusion surgery? a randomized clinical trial | Cochrane Library. doi:10.1002/central/CN-01647361
    - EUROSPINE Meetings 2018: abstracts | Cochrane Library. doi:10.1002/central/CN-01647379
  - **Trial protocol (n= 4)**
    - NL-OMON49688. The Impact of Cognitive Behavioral Therapy (CBT) on Treatment Outcome after Lumbar Spinal Fusion Surgery in Patients with High Pain Catastrophizing: a Two-center Randomized Controlled Trial of CBT vs. Education | Cochrane Library. doi:10.1002/central/CN-02719095
    - Lotzke H, Jakobsson M, Brisby H, et al. Use of the PREPARE (PREhabilitation, Physical Activity and exeRcisE) program to improve outcomes after lumbar fusion surgery for severe low back pain: a study protocol of a person-centred randomised controlled trial. BMC Musculoskelet Disord. 2016;17(1):349. Published 2016 Aug 18. doi:10.1186/s12891-016-1203-8
    - ISRCTN17115599. PREPARE (PREhabilitation, Physical Activity and ExeRcisE) persons with severe low back pain for an optimal functional outcome after lumbar fusion surgery | Cochrane Library. doi:10.1002/central/CN-01851402
    - Scarone P, Smeets AYJM, van Kuijk SMJ, van Santbrink H, Peters M, Koetsier E. A randomized controlled TRIal of cognitive BEhavioral therapy for high Catastrophizing in patients undergoing lumbar fusion surgery: the TRIBECA study. BMC Musculoskelet Disord. 2020;21(1):810. Published 2020 Dec 4. doi:10.1186/s12891-020-03826-w
  - **Different study design (n= 3)**
    - Scarone P, Van Santbrink W, Koetsier E, Smeets A, Van Santbrink H, Peters ML. The effect of perioperative psychological interventions on persistent pain, disability, and quality of life in patients undergoing spinal fusion: a systematic review. Eur Spine J. 2023;32(1):271-288. doi:10.1007/s00586-022-07426-1
    - Parrish JM, Jenkins NW, Parrish MS, et al. The influence of cognitive behavioral therapy on lumbar spine surgery outcomes: a systematic review and meta-analysis. Eur Spine J. 2021;30(5):1365-1379. doi:10.1007/s00586-021-06747-x
    - Gaudin D, Krafcik BM, Mansour TR, Alnemari A. Considerations in Spinal Fusion Surgery for Chronic Lumbar Pain: Psychosocial Factors, Rating Scales, and Perioperative Patient Education-A Review of the Literature. World Neurosurg. 2017;98:21-27. doi:10.1016/j.wneu.2016.10.124
  - **Different patient population (n= 2)**
    - Chavez JL, Porucznik CA, Gren LH, et al. The Impact of Preoperative Mindfulness-Based Stress Reduction on Postoperative Outcomes in Lumbar Spine Degenerative Disease: 3-Month and 12-Month Results of a Pilot Study. World Neurosurg. 2020;139:e230-e236. doi:10.1016/j.wneu.2020.03.186
    - Archer KR, Devin CJ, Vanston SW, et al. Cognitive-Behavioral-Based Physical Therapy for Patients With Chronic Pain Undergoing Lumbar Spine Surgery: A Randomized Controlled Trial. J Pain. 2016;17(1):76-89. doi:10.1016/j.jpain.2015.09.013
  - **Studies with overlapping cohorts coinciding with other studies (n= 3)**
    - Mansell G, den Hollander M, Lotzke H, Smeets RJEM, Lundberg M. A Person-Centred Prehabilitation Program based on Cognitive Behavioural Physical Therapy for patients scheduled for Lumbar Fusion surgery: A mediation analysis to assess fear of movement (kinesiophobia), self-efficacy and catastrophizing as mediators of health outcomes. Eur J Pain Lond Engl. 2022;26(8):1790-1799. doi:10.1002/ejp.2004
    - Rolving N, Nielsen CV, Christensen FB, Holm R, Bünger CE, Oestergaard LG. Does a preoperative cognitive-behavioral intervention affect disability, pain behavior, pain, and return to work the first year after lumbar spinal fusion surgery? Spine. 2015;40(9):593-600. doi:10.1097/BRS.0000000000000843
    - Rolving N, Nielsen CV, Christensen FB, Holm R, Bünger CE, Oestergaard LG. Preoperative cognitive-behavioural intervention improves in-hospital mobilisation and analgesic use for lumbar spinal fusion patients. BMC Musculoskelet Disord. 2016;17:217. doi:10.1186/s12891-016-1078-8
